# Emotion Naming Impedes Both Cognitive Reappraisal and Mindful Acceptance Strategies of Emotion Regulation

**DOI:** 10.1007/s42761-021-00036-y

**Published:** 2021-04-20

**Authors:** Erik C. Nook, Ajay B. Satpute, Kevin N. Ochsner

**Affiliations:** 1 Department of Psychology, Harvard University, William James Hall, 33 Kirkland St, Cambridge, MA 02138 USA; 2 Department of Psychology, Northeastern University, Boston, USA; 3 Department of Psychology, Columbia University, New York City, USA

**Keywords:** Emotion regulation, Cognitive reappraisal, Mindful acceptance, Emotion naming, Affect labeling

## Abstract

Friends and therapists often encourage people in distress to say how they feel (i.e., name their emotions) with the hope that identifying their emotions will help them cope. Although lay and some psychological theories posit that emotion naming should facilitate subsequent emotion regulation, there is little research directly testing this question. Here, we report on two experimental studies that test how naming the emotions evoked by aversive images impacts subsequent regulation of those emotions. In study 1 (*N* = 80), participants were randomly assigned into one of four between-subjects conditions in which they either (i) passively observed aversive images, (ii) named the emotions that these images made them feel, (iii) regulated their emotions by reappraising the meaning of images, or (iv) both named and regulated their emotions. Analyses of self-reported negative affect revealed that emotion naming impeded emotion regulation via reappraisal. Participants who named their emotions before reappraising reported feeling *worse* than those who regulated without naming. Study 2 (*N* = 60) replicated these findings in a within-participants design, demonstrated that emotion naming also impeded regulation via mindful acceptance, and showed that observed effects were unrelated to a measure of social desirability, thereby mitigating the concern of experimenter demand. Together, these studies show that the impact of emotion naming on emotion regulation opposes common intuitions: instead of facilitating emotion regulation via reappraisal or acceptance, constructing an instance of a specific emotion category by giving it a name may “crystalize” one’s affective experience and make it more resistant to modification.

**Supplementary Information:**

The online version contains supplementary material available at 10.1007/s42761-021-00036-y.

We are often encouraged to, “say how we feel,” when discussing life’s struggles with friends and therapists. This prompt reveals the widespread belief that identifying one’s emotions helps us cope. Although it is intuitive to believe that *emotion naming* (i.e., verbally identifying one’s emotions and vocalizing them aloud) facilitates *emotion regulation* (i.e., modifying one’s emotional responses; Gross, 1998, 2015), there is little empirical research testing this (e.g., Greenberg, 2004). Here, we examine how naming emotions impacts two different strategies for regulating one’s emotions: cognitive reappraisal by reinterpretation (i.e., rethinking the meaning of a stimulus to change its emotional impact; Mcrae et al., 2012; Ochsner et al., 2012) and mindful acceptance (i.e., seeing emotions as impermanent experiences that can be observed and let go; Kober et al., 2019; Kross et al., 2009).

There are three potential relationships between emotion naming and emotion regulation. First, emotion naming may facilitate regulation. Difficulties identifying and describing one’s emotions (called *alexithymia*; Sifneos, 1973) are associated with less frequent use of cognitive reappraisal (Swart et al., 2009), worse mental health (Leweke et al., 2011; Taylor et al., 1997), and poorer therapeutic outcomes (Ogrodniczuk et al., 2011). Additionally, classic developmental theories postulate that self-regulation requires the internalization of language (Luria, 1961; Meichenbaum, 1975), fostering the notion that children learn to regulate their emotions via the ability to identify their feelings (Kopp, 1989). Indeed, children with specific language impairment are perceived as less capable of regulating their emotions (Fujiki et al., 2002), and teenagers who struggle to specifically identify their emotions are at heightened risk of affective illnesses when exposed to stress (Nook, Flournoy et al., 2020; Starr et al., 2020). As such, emotion verbalization might bolster emotion regulation across the lifespan.

A second possibility is that emotion naming does not impact emotion regulation: naming and regulating may operate independently. Indeed, studies on *affect labeling* demonstrate that pairing aversive stimuli (e.g., pictures of frowning faces or disgusting insects) with affective labels (e.g., “sadness” or “cockroach”) reduces neural, psychophysiological, and self-reported indices of distress (Constantinou et al., 2014; Fan et al., 2019; Kircanski et al., 2012; Lieberman et al., 2007; Lieberman et al., 2011; Tabibnia et al., 2008; Torre & Lieberman, 2018). Ergo, labeling may itself regulate emotions. However, not all studies have found this effect, suggesting the presence of hidden moderators (Fitzpatrick et al., 2019; Matsuguma et al., 2020; McRae et al., 2010; Ortner, 2015). One study has investigated how affect labeling impacts the efficacy of *subsequent* cognitive emotion regulation. Using one trial per condition, this study found that generating labels for one’s emotional responses to audio vignettes led to reduced self-reported negative affect following emotion regulation in healthy participants, but this did not occur for participants with borderline personality disorder (Fitzpatrick et al., 2019). Given these mixed results, additional research on how naming impacts both emotion and emotion regulation is needed.

Third, emotion naming might impede emotion regulation. According to constructionist theories of emotion, *emotions* arise when bodily sensations and other sensory signals are categorized using emotion concepts (Barrett, 2006, 2017). This theory draws support from evidence that emotion words and concepts play a central role in transforming raw undifferentiated “core affect” into discrete emotional experiences (Barrett, 2006, 2017; Lindquist et al., 2015; Nook et al., 2015; Satpute et al., 2016; Satpute et al., 2020). Based on this theory, we posit that emotions—once “constructed”—may be more resistant to change than undifferentiated affect. In other words, emotion naming might “crystallize” one’s affective state, making it more difficult to modify through emotion regulation. It is also possible that naming one’s emotions might deplete resources that could otherwise be used for emotion regulation. Cognitively demanding activities can reduce motivation for subsequent tasks (Inzlicht & Schmeichel, 2012), and given that both categorizing (Lieberman et al., 2007; Satpute et al., 2013) and regulating (Buhle et al., 2014) emotions recruit prefrontal regions implicated in effortful control, it is possible that emotion naming could also impede regulation by depleting motivation to re-engage control processes for subsequent regulation.

Here, we adjudicate between these hypotheses through two studies that asked participants to interact with aversive images either by passively viewing them, naming their emotions, regulating their emotions, or both naming and regulating their emotions. These factorial designs tested whether naming facilitates, impedes, or has no impact on subsequent regulation.

## Study 1

### Method

#### Participants

Eighty-one individuals provided informed consent to participate in study 1. One participant did not complete the experiment. Hence, analyses include data from 80 participants (68.75% female, 1 did not disclose gender, age range = 18–33, *M*_age_ = 20.91, *SD*_age_ = 3.41). All participants were fluent in English and received US$12/h for their time. Participants were evenly divided into 4 between-participants conditions. An initial pilot study (separate from data presented here) of 5 participants in each of the four conditions revealed a large difference between the *Regulate* and *Name and Regulate* conditions (Cohen’s *d* = 1.24). Given that this was the contrast that was most relevant to the study’s research questions, we used this estimated effect size to plan the sample size of the full study. A power analysis suggested that 12 participants per condition were required to detect an effect of this size at 80% power when α = .05. We increased this target to 20 per condition prior to initiating data collection to provide ample power to detect differences across all 4 conditions. We did not perform a literature-based power analysis given that no prior studies had investigated the effect of emotion naming on emotion regulation at the time of study design.

#### Experimental Paradigm and Procedure

**Fig. 1:**
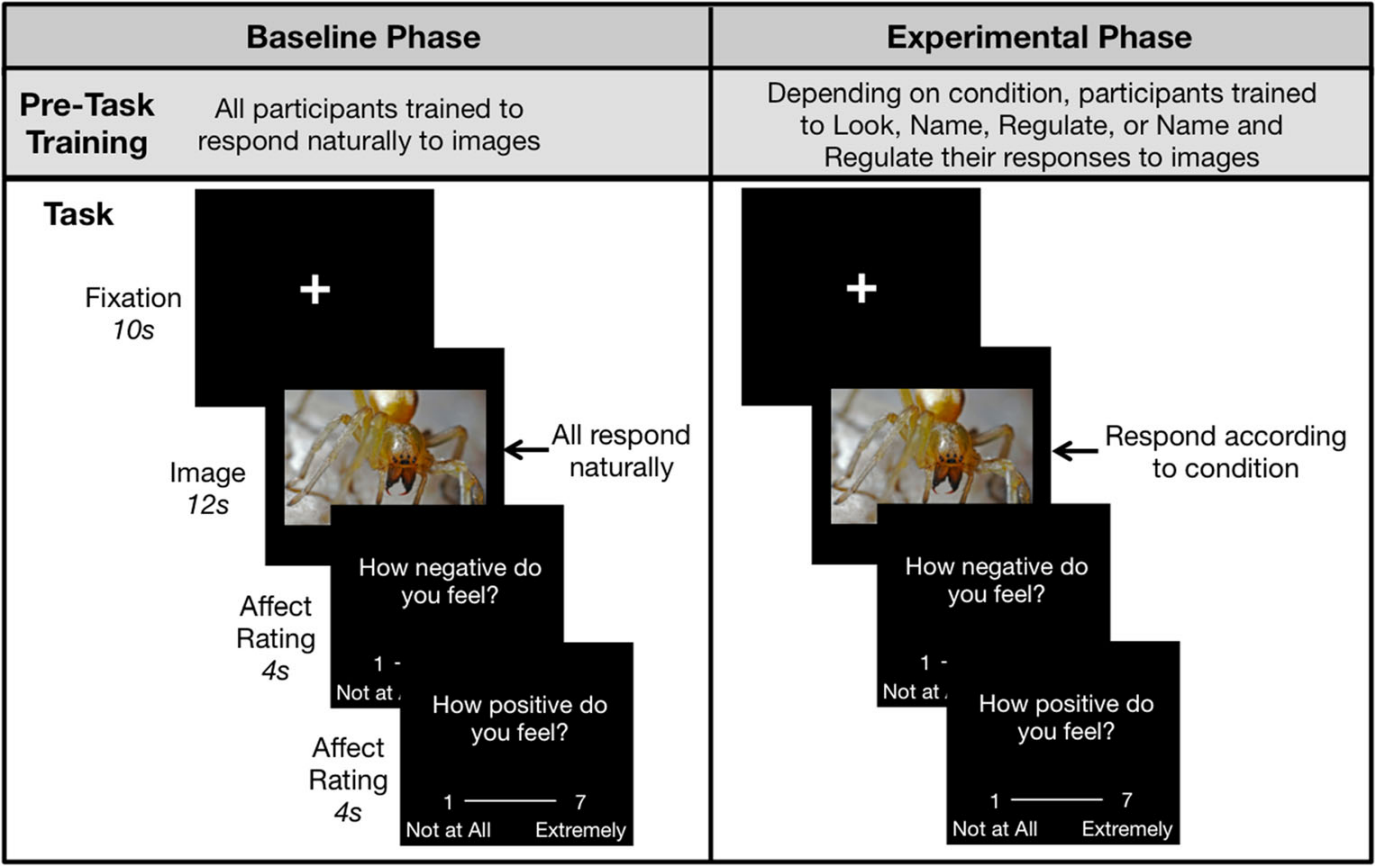
Study 1 task schematic depicting both baseline and experimental phases, with trainings preceding each phase. Participants first completed a baseline phase, in which they responded naturally to a set of 24 negative IAPS images and rated how positive and negative each made them feel. They then completed an experimental phase. Instructions for the experimental phase varied across between-participants conditions. Some participants again responded naturally to the images (*Look* condition), some named the most dominant emotion that the image made them feel (*Name* condition), some regulated their response to the images (*Regulate* condition), and some both named and regulated their emotions (*Name and Regulate* condition). Participants again rated now positive and negative they felt after each image

For both experiments, we adapted a commonly used cognitive reappraisal paradigm (Buhle et al., 2014; Nook et al., 2017; Nook, Vidal Bustamente et al., 2020; Ochsner et al., 2002) to examine how emotion naming impacts emotion regulation (Fig. 1). In particular, we focused on two variants of reappraisal known as reinterpretation (McRae et al., 2012; Ochsner et al., 2012) and mindful acceptance (Hayes et al., 2006; Kross et al., 2009). Although there are many methods for regulating one’s emotions, we focused on these strategies because they are (i) explicit strategies that can be employed or not employed in response to any given stimulus (thereby making them manipulable across conditions), (ii) easily studied through well-established paradigms, and (iii) similar to emotion regulation strategies utilized in clinical settings (Goldin et al., 2012; Hayes et al., 2006).

We provide a few notes on terminology before proceeding with the emotion regulation task description. Constructionist theories often refer to *affect* as the undifferentiated, low-dimensional sensations people experience at all times, and *emotions* as the product of parsing affect into a specific emotion type using an emotion concept (Barrett, 2006, 2017; Russell, 2003; Russell & Barrett, 1999). Our use of these terms follows these theoretical definitions. We use the phrase *emotion regulation* to refer to any instance in which people set goals to change how they feel (Gross, 1998) even though individuals in our studies may have been regulating undifferentiated *affect* (i.e., in conditions where they did not name their emotions). We do this because our methods and theoretical framework draw directly from Gross’s (1998, 2015) model of emotion regulation. Relatedly, Gross and Barrett (2011) argue that emotion construction and emotion regulation may or may not be identical processes. Indeed, both constructing and regulating emotions can involve selecting appraisals of a stimulus that shift one’s conceptualization (and thus emotional reaction) to that stimulus. Nonetheless, to answer this paper’s primary research questions, we found it valuable to distinguish conceptualizing and verbalizing one’s emotional reaction to a stimulus (i.e., “naming”) from changing one’s cognitions about a stimulus to modify one’s emotional response (i.e., “regulating”). As such, we refer to these tasks using different terms, even if they may involve overlapping cognitive processes (see also Braunstein et al., 2017). Finally, we refer to our manipulation of generating and vocalizing a name for one’s current emotions as *emotion naming* because it differs in focus and method from much of the work on *affect labeling*, which typically instead pairs images with provided labels that may or may not refer to the participant’s own emotional states (e.g., Lieberman et al., 2007, 2011).

Following informed consent, the procedure for study 1 involved two phases. The first *baseline phase* asked participants to simply observe and report on their affective responses to 24 negative images (full instructions for both studies are provided in the Supplemental Materials). We included this phase to establish baseline reactions to images against which we could compare changes in emotional response as a function of emotion naming and/or regulation. Concretely, we subtracted these baseline reactions away from participants’ reactions when they were naming and/or regulating their responses to the same images in the following experimental phase of the study. Images were drawn from the International Affective Picture Set (IAPS; Lang et al., 2008). IAPS IDs for images from both studies are provided in the Supplemental Materials. Valence norms for all images were moderately-to-extremely negative (range = 1.6–3.95 on a 1–9 scale where lower numbers are more negative, *M*_valence_ = 2.68, *SD*_valence_ = 0.63), and arousal norms were moderately-to-highly arousing (range = 4.14–7.09 on a 1–9 scale where higher numbers are more arousing, *M*_arousal_ = 5.69, *SD*_arousal_ = 0.78). On each trial, participants passively viewed images and then used the keyboard to rate how much negative and positive affect each made them feel (1 = *not at all*, 7 = *extremely*). Negative and positive affect rating order was randomized across trials, but negative affect preceded positive affect for one half of the images. The use of separate unipolar scales rather than a single bipolar scale was used to capture the possibility of participants feeling high levels of both positive and negative affect (Kron et al., 2013).

Participants then completed the *experimental phase* in which they viewed the same 24 images a second time. Before this phase, participants were randomly assigned to one of four between-participants conditions that produced a 2 [Naming: No Naming vs. Naming] × 2 [Regulating: No Regulating vs. Regulating] factorial design. The conditions were: (i) *Look* (no naming + no regulating), (ii) *Name* (naming + no regulating), (iii) *Regulate* (no naming + regulating), and (iv) *Name and Regulate* (naming + regulating).

In the *Look* condition, participants were given the same instructions as the baseline phase. They simply observed the images a second time. This condition allowed us to capture how much affect ratings changed merely due to re-exposure. In the *Name* condition, participants said aloud the most dominant emotion that they felt while each image was on the screen. Comparing *Name* and *Look* conditions allowed us to test the affective impact of emotion naming alone. In the *Regulate* condition, participants regulated their emotional responses to images using cognitive reappraisal. Specifically, participants employed the reinterpretation variant of reappraisal in which they silently created a story or context for each image that made it less aversive (Gross, 2015; McRae et al., 2012; Ochsner et al., 2012). Finally, in the *Name and Regulate* condition, participants received both the *Name* and *Regulate* instructions (i.e., they verbally identified the most dominant emotion an image made them feel and then regulated their emotional response to the image). Comparing the *Regulate* and the *Name and Regulate* conditions allowed us to test how naming emotions impacted emotion regulation.

The experimenter verified participants’ comprehension of and compliance with task instructions before each phase through a series of practice trials. Verbal responses in both the *Name* and *Name and Regulate* conditions were recorded by microphone, and recordings were checked for compliance. We adopted an inclusive approach to this task, allowing participants to naturalistically name their emotions using any term they generated (see Supplemental Materials). However, responses that clearly could only apply to the content of the image rather than an emotional response (e.g., “bug,” “cut”) were deemed non-compliant. Across participants in the *Name* and *Name and Regulate* conditions, non-compliant naming was rare (i.e., the mean number of trials for which no response was given, the response was unintelligible, or the response referred to image contents rather than an emotion was 1.05 of 24). No participant exceeded our a priori exclusion threshold of one third non-compliant trials. We excluded affect ratings for trials in which participants did not provide compliant emotion names from all analyses. Excluding these trials did not affect the significance of any results presented in the manuscript.

#### Analyses

Within-person correlations using the *psych* package (Revelle, 2016) revealed—as expected—that negative and positive affect ratings shared a strong negative correlation across trials, *r*_baseline_ = − .53, *r*_experimental_ = − .49, *p*s < .001. Hence, we combined these ratings into a single measure of *unpleasant affect* [unpleasant affect rating for each trial = mean((negative affect rating) + (8 − positive affect rating))]. Trials on which participants did not provide a response to either the negative or the positive affect rating were excluded from analyses. We then computed each participant’s *change in unpleasant affect* (*Δ unpleasant affect*) by subtracting their mean baseline-phase unpleasant affect rating from their mean experimental-phase unpleasant affect rating. This comprised our primary dependent variable of interest. Although the trial-level correlation between negative and positive affect was strong, visualization of trial-level responses revealed that positive affect ratings clustered at the floor of the rating scale—likely because all images were negative (see Supplemental Materials for distributions). As such, the restricted range in positive affect ratings may have even *reduced* the magnitude of the trial-level correlation between positive and negative affect ratings. Although this further supports the decision to collapse these ratings, we also present analyses of Δ negative and Δ positive affect (which largely reflect results of Δ unpleasant affect analyses) in the Supplemental Materials for completeness.

We analyzed Δ unpleasant affect using a 2 [Naming] × 2 [Regulating] ANOVA. As outlined in the introduction, evidence for the three hypothesized relationships between emotion naming and emotion regulation hinge on the presence and direction of the interaction between these factors. We also planned two additional sets of analyses. First, to fully unpack interactions, we conducted independent-samples *t* tests comparing Δ unpleasant affect between (i) the *Regulate* and *Name and Regulate* conditions and (ii) the *Look* and *Name* conditions. These analyses allowed us to assess whether (i) regulation was more or less successful if paired with naming and (ii) whether naming in itself altered affect. Second, we conducted 4 one-way *t* tests to examine whether each condition’s Δ unpleasant affect differed significantly from 0. These analyses tested whether participants’ affect differed between the baseline and experimental phases, thereby allowing us to observe whether instructions for the experimental phase of each condition significantly affected their emotional experience compared to baseline.

A coding error caused images to be displayed for inconsistent times in the experimental phase. After the 12th trial, images were displayed for less than the intended 12 s (decreasing to approximately 10 s). No participants reported noticing the error. We report data from all trials of each condition because the significance of unpleasant affect analyses is identical when we use ratings only from the first 12 trials of the experimental phase (i.e., when we ensure that the length of image exposure is consistent across the two phases). We report 95% confidence intervals (CIs) for effect sizes of *t* tests and 90% CIs for effect sizes of ANOVAs (*F* tests) following the guidance of Lakens (2013). Data for both studies can be found at https://osf.io/59jqd/.

### Results

**Fig. 2:**
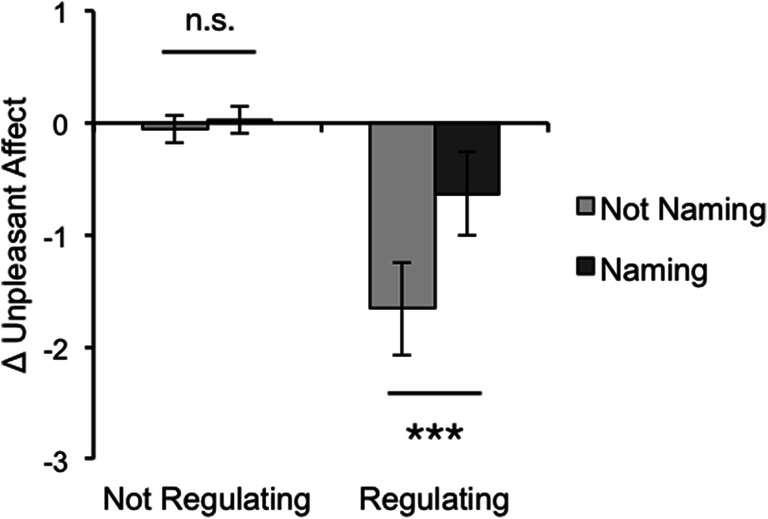
Study 1 results. Mean change in unpleasant affect from baseline to experimental phase, split by condition. The non-significant difference between *Look* (not naming + not regulating) and *Name* (naming + not regulating) conditions suggests that merely naming emotions does not change affective responses to images. The significant difference between the *Regulate* (not naming + regulating) and *Name and Regulate* (naming + regulating) conditions suggests that naming impedes regulation. Error bars represent 95% CIs. n.s. *p* > .05, ***p < .001

Data supported the third hypothesized relationship: Emotion naming impeded emotion regulation via reinterpretation (Fig. 2). We observed a main effect of regulating on Δ unpleasant affect such that participants felt better after regulating their emotions (*M* = − 1.15, *SD* = 0.98) compared to not regulating (*M* = − 0.01, *SD* = 0.26), *F*(1,76) = 65.22, *p* < .001, η_*p*_^2^ = .46, 90% CI = [.32, .56]. We also observed a main effect of naming on Δ unpleasant affect such that participants felt less negative after not naming their emotions (*M* = − 0.86, *SD* = 1.04) compared to naming (*M* = − 0.30, *SD* = 0.68), *F*(1,76) = 15.74, *p* < .001, η_*p*_^2^ = .17, 90% CI = [.06, .29]. However, these effects were qualified by a significant interaction between naming and regulating, *F*(1,76) = 11.17, *p* = .001, η_*p*_^2^ = .13, 90% CI = [.03, .25].

Planned *t* tests revealed that emotion naming diminished the impact of emotion regulation: Δ unpleasant affect rating scores decreased more strongly when participants *Regulated* their emotional responses to images (*M* = − 1.66, *SD* = 0.89) compared to when they *Named and Regulated* their emotions (*M* = − 0.63, *SD* = 0.80), *t*(38) = − 3.82, 95% CI of difference in means = [− 1.57, − 0.48], *p* < .001, *d* = − 1.21. Additionally, naming alone did not downregulate affect: the average Δ unpleasant affect ratings was statistically identical when participants *Looked* at images a second time (*M* = − 0.06, *SD* = 0.26) and when they *Named* the emotions these images aroused (*M* = 0.03, *SD* = 0.26), *t*(38) = − 1.07, 95% CI = [− 0.25, 0.08], *p* = .293, *d* = − 0.34.

Finally, one-way *t* tests revealed that Δ unpleasant affect did not differ significantly from 0 in either the *Look* condition, *t*(19) = − 0.98, *p* = .339, 95% CI = [− 0.18, 0.06], *d* = 0.22, or in the *Name* condition, *t*(19) = 0.52, 95% CI = [− 0.09, 0.15], *p* = .606, *d* = 0.12. This suggests that neither re-exposure nor emotion naming changed affective responses to images. However, Δ unpleasant affect was significantly lower than 0 in both the *Regulate*, *t*(19) = − 8.29, *p* < .001, 95% CI = [− 2.08, − 1.24], *d* = − 1.85, and *Name and Regulate* conditions, *t*(19) = − 3.55, *p* = .002, 95% CI = [− 1.01, − 0.26], *d* = − 0.79, suggesting that participants successfully modulated their affect in both conditions.

## Study 2

Study 1 provided initial evidence that naming emotions disrupts emotion regulation via reinterpretation, challenging common intuitions about the impact of emotion naming on emotion regulation. However, four potential concerns must be addressed before drawing this conclusion. First, a timing confound may explain these results. Participants in the *Regulate* condition had 12 seconds to reinterpret images, whereas participants in the *Name and Regulate* condition had 12 seconds to both name emotions and reinterpret images. Hence, participants in the *Regulate* condition may have been more successful at regulating simply because they had more time to do so. Second, study 1 merely demonstrated that emotion naming impeded cognitive reappraisal by reinterpretation, but naming may facilitate other reappraisal strategies. Third, Study 1 did not test whether results were influenced by participants’ motivation to respond in socially desirable ways (e.g., to conform to their expectations of the study’s predicted results). Fourth, there were only 12 trials for which the image was displayed for a full 12 s in the experimental phase. Although results are identical when restricting analyses to full-length trials, stability of these measures may be low.

Study 2 addressed these concerns. We altered the design to replicate results within-person and to control the duration of time participants spent naming and regulating in each condition. We also added a between-participants manipulation of the type of emotion regulation strategy participants used: *reinterpretation* or *acceptance*. Acceptance has its origin in mindfulness practices and involves viewing emotions as impermanent mental experiences that are accepted as they are (Hayes et al., 2006). Whereas reinterpretation involves changing one’s understanding of the stimuli that evoke emotions, acceptance involves changing one’s attitudes toward emotions themselves. As such, naming an emotion might facilitate acceptance by increasing awareness and understanding of one’s emotional experience, qualities that are considered helpful to regulation in theories of both mindfulness and self-determination (Brown et al., 2007; Deci & Ryan, 2012; Gratz & Tull, 2010). Finally, we added a social desirability questionnaire to test whether participants’ responses tracked their sensitivity to behave in socially desirable ways.

### Method

#### Participants

Seventy-two participants consented to participate in Study 2. One participant did not complete the experiment, and 11 participants failed to adequately follow emotion-naming instructions (i.e., for more than one third of the trials in either the *Name* or *Name and Regulate* conditions, their response was absent, unintelligible, or about the content of the image rather than their emotions). Hence, analyses include data from 60 viable participants (70.00% female, age range = 18–31, *M*_age_ = 20.53, *SD*_age_ = 2.71). Excluding the 11 participants who did not comply with naming instructions did not affect the significance of any results presented in the manuscript. For remaining participants, trials with unusable naming data were rare (*M* = 2.7 of 40 naming trials). We again excluded these trials from analyses, and doing so did not affect the significance of any results presented in the manuscript. All participants were fluent in English and received US$12/h for their time.

A power analysis using the data from study 1 suggested that only 8 participants were required within each between-participants condition to detect a within-participants difference between the *Regulate* and the *Name and Regulate* conditions (*d* = 1.21) with 80% power. However, to provide a fair test of possible differences between the *Look* and *Name* condition, we increased the sample size and sought 30 participants in each between-participants condition (60 participants total) to allow for adequate power to detect medium-sized effects (Cohen, 1988). We recruited extra participants (i.e., 12 more for a total of 72) to allow us to replace those who failed to adequately follow emotion naming instructions, as described above.

#### Paradigm and procedure

**Fig. 3:**
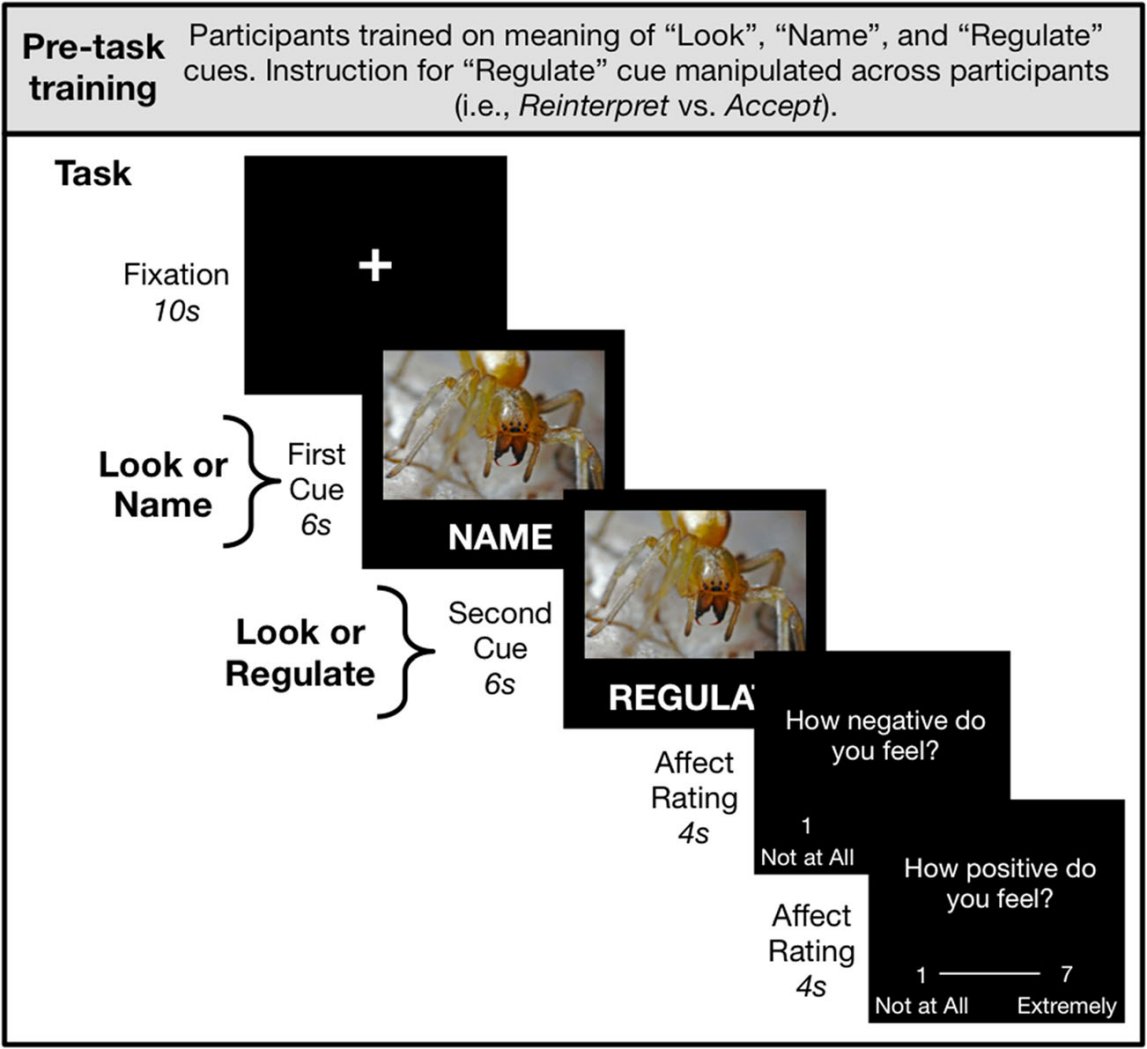
Study 2 task schematic including pre-task training. Participants completed 80 trials of a task that implemented the study 1 Naming × Regulating design within-participants and introduced a new between-participants variable of *emotion regulation strategy*. On each trial, participants saw a negative IAPS image along with a combination of 2 cues presented under the image for 6 s each. The “Look” cue indicated that participants should passively observe the image, the “Name” cue indicated that participants should say aloud the most dominant emotion they felt, and the “Regulate” cue indicated that participants should regulate their emotions. Cue combinations reproduced the Study 1 design within participants: *Look* trial = “Look” + “Look” cues, *Name* trial = “Name” + “Look” cues, *Regulate* trial = “Look” + “Regulate” cues, and *Name and Regulate* trial = “Name” + “Regulate” cues. Additionally, the strategy participants used to regulate their emotions was manipulated between participants: Some participants were instructed to *reinterpret* the meaning of the image (as in study 1), and others were instructed to mindfully *accept* their emotions

We adapted the paradigm of Study 1 in three ways (Fig. 3): (i) we collapsed the study 1 Naming × Regulating between-participants design into a within-participants design, (ii) we removed the baseline phase to reduce participant burden given the increased number of images needed for the within-participants design, and (iii) we added a between-participants manipulation such that some participants regulated their emotions by reinterpreting the meaning of the images as in study 1, whereas other participants regulated by mindfully accepting the emotions they were feeling (Kross et al., 2009).

As in study 1, participants interacted with images and then rated how each made them feel. However, in this study, image exposure was divided into two 6-s windows. An instruction (“Look,” “Name,” or “Regulate”) appeared under the image for the first 5.5 s of these windows (see next paragraph for description of second window). All participants were instructed that the cue word “Look” indicated that they should simply observe and respond naturally to the image, and the word “Name” indicated that they should say aloud the most dominant emotion that the image made them feel. The meaning of the “Regulate” cue varied across our between-participants manipulation of *emotion regulation strategy*. Participants in the *Reinterpret* condition (*N* = 29) were instructed that they should reinterpret the meaning of the image to feel better about it (as in study 1), but participants in the *Accept* condition (*N* = 31) were instructed to “try to make yourself feel better about the image by accepting whatever it makes you feel… Whatever you feel is just an emotion; it cannot harm you and it will soon pass. Make yourself feel better by acknowledging what you feel and letting it go.”

For one quarter of trials, the cue word “Look” was presented for both the first and second 6-s windows of the image exposure. This corresponds to the *Look* (no naming + no regulating) condition of study 1. For one quarter of trials, the cue for the first window was “Name” and the second cue was “Look,” corresponding to the *Name* (naming + no regulating) condition. For another quarter of trials, the cues were “Look” and “Regulate,” corresponding to the *Regulate* (no naming + regulating) condition. The final quarter of trials involved the cues to “Name” and “Regulate,” corresponding to the *Name and Regulate* (naming + regulating) condition. After interacting with the image by implementing the instructions of the two cues, participants rated their positive and negative affect using the same procedure as Study 1. Participants completed 80 trials grouped into two runs of 40 trials with a short break between runs. Trial order was randomized across participants.

Four lists of negative IAPS images were created to have equivalent normed valence ratings, *F*(3, 76) = 0.08, *p* = .972 (*t* tests comparing each set to all others: *p*s > .646), and arousal ratings, *F*(3, 76) = 0.14, *p* = .934, (*t* tests comparing each set to all others: *p*s > .507). We counterbalanced the assignment of image lists to each within-participants condition (i.e., *Look*, *Name*, *Regulate*, and *Name and Regulate*) across participants.

#### Social Desirability Questionnaire

Participants completed the Marlowe-Crowne Social Desirability Scale (Crowne & Marlowe, 1960) following the experimental task. Participants responded to 33 true/false questions that measured their motivation to respond in ways that are socially desirable (e.g., responding “true” to “I never hesitate to go out of my way to help someone in trouble” or “false” to “I like to gossip at times”). Items are scored as 1 (socially desirable response given) or 0 (non-socially desirable response given). Item scores are summed to produce totals ranging between 0 and 33, with higher scores representing more socially desirable responding. This scale is commonly used to measure and control for response bias in studies involving self-report methods (Larson, 2019; van de Mortel, 2008; Vesely & Klöckner, 2020). The scale showed strong reliability in the current study (Cronbach’s α = .80).

#### Analyses

A within-subjects correlation again suggested that negative and positive affect ratings were inversely correlated across trials, *r* = − .55, *p* < .001. Trial-level positive affect ratings were again clustered at floor, potentially reducing their correlation with negative affect ratings (see Supplemental Materials). Hence, we computed each participant’s mean unpleasant affect rating for images in each condition, as in study 1. We analyzed unpleasant affect ratings using a 2 [Naming: naming vs. not naming] × 2 [Regulating: regulating vs. not regulating] × 2 [Emotion Regulation Strategy: reinterpret vs. accept] mixed ANOVA. Analyses of negative and positive affect largely reflect analyses of unpleasant affect, but we present them in the Supplemental Materials for completeness. As in study 1, we unpacked interactions using planned t-tests. Within each regulation strategy condition, we compared unpleasant affect ratings for (i) the *Regulate* and the *Name and Regulate* conditions to assess whether naming emotions before regulating them impacted regulation success and (ii) the *Look* and *Name* conditions to assess whether merely naming emotions altered affective responses to images.

To test whether participants’ self-reported emotion ratings may have been influenced by socially desirable responding, we tested for correlations between social desirability scores on the Marlowe-Crowne Scale and participants’ average unpleasantness ratings in each condition. We also specifically tested whether social desirability scores correlated either with the impact of naming alone on participants’ emotions (i.e., by computing the difference between participants’ average unpleasantness rating following *Look* trials vs. *Name* trials) or with the impact of naming on regulation (i.e., by computing the difference in participants’ unpleasantness ratings following *Regulate* vs. *Name and Regulate* trials). We conducted these correlations both across all subjects and separately within the *Reinterpret* and *Accept* conditions.

### Results

**Fig. 4:**
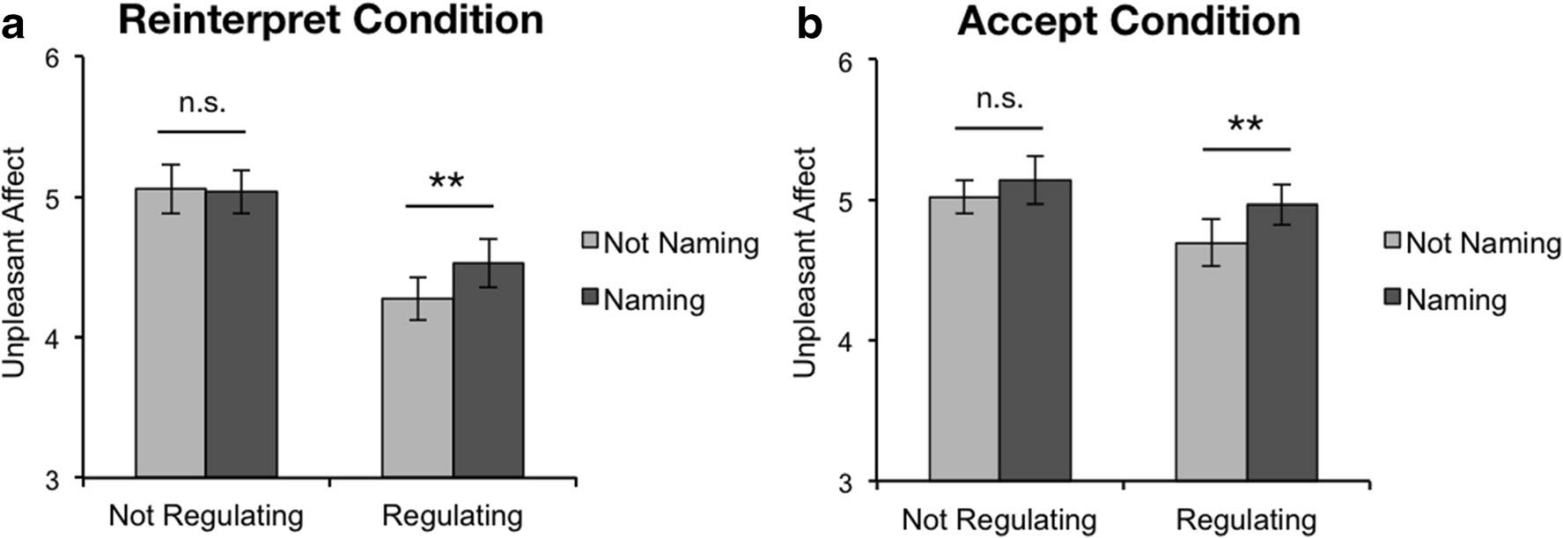
Study 2 results. Mean unpleasant affect ratings, split by condition. As in study 1, the significant difference between the *Regulate* (regulating + not naming) and *Name and Regulate* (regulating + naming) trials in both the Reinterpret and Accept conditions suggests that naming emotions impedes both emotion regulation strategies. Additionally, the non-significant difference between Look (not regulating + not naming) and Name (not regulating + naming) trials suggests that naming emotions does not alter emotional responses to images. Error bars represent 95% CIs adjusted for within-participants variance (Morey, 2008). n.s. p > .05, **p < .01

The pattern of results found in study 1 for the *Reinterpret* condition was replicated, and the same pattern was found in the *Accept* condition as well (Fig. 4). An ANOVA across all data revealed a main effect of regulation on unpleasant affect ratings such that participants felt less negative after regulating their emotions (*M* = 4.62, *SD* = 0.91) compared to not regulating (*M* = 5.06, *SD* = 0.78), *F*(1,58) = 33.40, *p* < .001, η_*p*_^2^ = .37, 90% CI = [.20, .49]. There was also a main effect of naming such that participants felt less negative on trials when they did not name their emotions (*M* = 4.76, *SD* = 0.87) compared to when they did name their emotions (*M* = 4.92, *SD* = 0.88), *F*(1,58) = 16.04, *p* < .001, η_*p*_^2^ = .22, 90% CI = [.08, .35]. However, these main effects were again qualified by a significant interaction between naming and regulating, *F*(1,58) = 8.65, *p* = .005, η_*p*_^2^ = .13, 90% CI = [.02, .26].

Within this omnibus ANOVA, we observed neither a main effect of emotion regulation strategy on unpleasant affect, *F*(1,58) = 1.41, *p* = .240, η_*p*_^2^ = .02, 90% CI = [0, .12], nor an interaction between regulation strategy and naming, *F*(1,58) = 0.98, *p* = .327, η_*p*_^2^ = .02, 90% CI = [0, .10]. However, there was a significant interaction between emotion regulation strategy and regulating, *F*(1,58) = 6.60, *p* = .013, η_*p*_^2^ = .10, 90% CI = [.01, .23] indicating that regulating by reinterpretation led to greater reductions in unpleasant affect (mean difference between regulating conditions and non-regulating conditions in reinterpret condition = − 0.65, *SD* = 0.62) compared to regulating by acceptance (*M* = − 0.25, *SD* = 0.58). Critically, however, there was no 3-way interaction between naming, regulating, and emotion regulation strategy, suggesting that emotion naming impacted emotion regulation similarly in both the *Reinterpret* and *Accept* conditions, *F*(1,58) = 0.74, *p* = .394, η_*p*_^2^ = .01, 90% CI = [0, .09].

Parallel to study 1 analyses, we used a series of planned *t* tests to unpack the interaction between naming and regulating within each between-participants condition. We again found that naming emotions impeded emotion regulation: Unpleasant affect was significantly lower following *Regulate* trials than *Name and Regulate* trials, both within the *Reinterpret* condition, *t*(28) = − 3.41, *p* = .002, 95% CI = [− 0.41, − 0.10], *d* = − 0.63, and the *Accept* condition, *t*(30) = -3.23, *p* = .003, 95% CI = [− 0.44, − 0.10], *d* = − 0.58. Likewise, we found that naming emotions did not significantly affect emotional experience: Unpleasant affect did not differ significantly between *Look* and *Name* trials both within the *Reinterpret* condition, *t*(28) = 0.25, *p* = .805, 95% CI = [− 0.13, 0.17], *d* = 0.05, and the *Accept* condition, *t*(30) = − 1.83, *p* = .078, 95% CI = [− 0.26, 0.01], *d* = − 0.33. Even though a trending effect of naming emerged in the *Accept* condition, the difference in means was the opposite of prior studies on affect labeling: Participants felt slightly *worse* (at a trending level of significance) after naming their emotions.

We observed no evidence that these results were influenced by social desirability, replicating Ochsner et al. (2002). Social desirability scores from the Marlowe-Crowne Social Desirability Scale did not correlate with unpleasantness ratings in any condition, either across all participants or separately in the reinterpret and accept conditions, *p*s > .276. Furthermore, social desirability scores did not correlate with the impact of naming on participants’ affect (i.e., there was no correlation between social desirability and the difference in participants’ unpleasantness ratings between *Look* trials and *Name* trials, *p*s > .309), nor was social desirability related to the impact of naming on regulating (i.e., there was no correlation between social desirability and the difference in participants’ unpleasantness ratings between *Regulate* trials and *Name and Regulate* trials, *p*s > .405) either across all participants or in the *Reinterpret* or *Accept* conditions separately.

## General Discussion

Contrary to lay and some psychological intuitions, naming emotions impeded emotion regulation in two studies. In study 1, participants who named their emotions before regulating reported feeling worse than participants who regulated their affect without naming. Study 2 replicated this result in a within-participants design and further showed that emotion naming diminished both reinterpretation and acceptance regulation strategies. Additionally, Study 2 results were not related to a measure of social desirability, mitigating concerns that results are due to experimenter demand. These results have important implications for basic and applied psychological theories.

Current emotion theories are unclear on how naming emotions should impact emotion regulation. Because cognitive control theories postulate that closely monitoring a system’s current state facilitates regulation of that system (Ullsperger et al., 2014), one could hypothesize that clarifying one’s initial emotional state by giving it a name would similarly facilitate emotion regulation. However, our data opposed this hypothesis, prompting questions as to why naming might impede regulation and how this finding squares with research showing that difficulties labeling emotions is associated with psychopathology (e.g., Taylor & Bagby, 2004). One possible explanation is that emotion naming “crystallizes” affect, consolidating appraisals corresponding to the chosen emotion word and thereby limiting one’s ability to generate alternative appraisals of a stimulus. If discrete emotions arise when ambiguous affective states are parsed using emotion concepts (Barrett, 2006, 2017), it is possible that finding and applying an emotion concept requires elaborating on one’s initial appraisals of a stimulus. Similar to depth of processing effects in memory research (Craik & Tulving, 1975) or the idea that speaking aligns language and thought (Satpute et al., 2016; Slobin, 1987), selecting a fitting emotion name to categorize one’s affect could entrench appraisals that align with the emotion category chosen. If so, emotion naming would hinder modification of these appraisals and thereby inhibit cognitive emotion regulation. A second possible explanation is that emotion naming may be taxing and reduce motivation to engage in subsequent regulation (Inzlicht & Schmeichel, 2012). Third, it’s likely that *how*—not just *whether—*individuals name their emotions influences emotion regulation. Indeed, more elaborative emotion naming leads participants to plan less optimal regulatory strategies (Vine et al., 2019), and meta-analyses suggest that the types of words used in neuroimaging tasks influences amygdala responses to aversive images (Brooks et al., 2017). Similarly, timing may play a role: Naming might crystallize affect in the short term but increase understanding of one’s emotions and thereby facilitate self-regulation in the long term (Hoemann et al., 2019; Kashdan et al., 2015). Although the current study allows for the conclusion that, *on average*, emotion naming impairs immediate regulation, future research on moderators of this effect is needed.

In our studies, emotion naming did not itself reduce negative affect. Although this is inconsistent with prior work on affect labeling (Torre & Lieberman, 2018), variations in task paradigms provide one possible explanation for these divergent results. Labeling affect can take many forms, and specific implementations could have divergent consequences. For instance, labeling aspects of aversive *stimuli* may be more effective at down-regulating emotion than labeling one’s *emotional experiences* (Fitzpatrick et al., 2019; McRae et al., 2010; Ortner, 2015). Additionally, *selecting* from labels that are provided (as is done in many studies) may lead to less cognitive/elaborative processing of the stimuli than *generating* one’s own labels (as was done in the current study). Further work is needed to identify the factors that influence how verbalizing emotions impacts emotional experience.

In terms of applications, our findings challenge the clinical intuition that therapeutic interventions will be more effective if patients first identify what they are feeling (Greenberg, 2004). Instead, naming emotions may not be advised in situations where regulation follows immediately after labeling. Indeed, thoroughly describing one’s feelings immediately following traumatic events is actually associated with *worse* outcomes (Mayou et al., 2000). That said, Fitzpatrick et al. (2019) found that labeling one’s emotions synergistically interacted with subsequent emotion regulation in healthy controls. Although it is unclear why our results differ from this study, it is possible that differences in stimuli (audio vignettes vs. images), labeling method (providing a list of labels vs. allowing participants to generate labels), or other aspects of the tasks (e.g., using > 4 trials) might explain these divergent results. As such, further research is needed to clarify the impact of emotion naming, especially in clinical settings. For instance, it is possible that emotion naming may be helpful when regulation takes place over long periods of time, when people have the opportunity to incorporate their emotions into a coherent narrative (Pennebaker & Chung, 2007), when people verbally express their emotions to others (Reeck et al., 2016; Zaki & Williams, 2013), or when it is used to make sense of complex affective experiences that arise in one’s life rather than relatively more simple reactions to aversive images. Examining these factors is crucial to charting the boundary conditions of how emotion naming influences emotion regulation.

Overall, findings from these studies extend emotion theories by revealing that emotion naming can impede the efficacy of two emotion regulation strategies. Understanding how language shapes emotion regulation is an important frontier of scientific discovery, as language provides both a ubiquitous measure of emotion and a clear point of intervention to influence emotions. Clarifying how and why naming impacts regulation represents an exciting opportunity to advance both basic understanding of emotional experience and clinical interventions.

## Supplementary Information

ESM 1 (DOCX 169 kb)

## Additional Information

### Data and Analytic Code Availability

Data and analytic code for both studies are available at https://osf.io/59jqd/?view_only=7da74b9cc7744c48a9e308e014cc3ce0.
