# Supplementary material for: Emotion Naming Impedes Both Cognitive Reappraisal and Mindful Acceptance Strategies of Emotion Regulation
Source: Affect Sci. 2021 Apr 20;2(2):187–98. doi: 10.1007/s42761-021-00036-y (PMC9383041; doi:10.1007/s42761-021-00036-y)
Supplement: Supplementary file 1 — (DOCX 169 kb) [file 42761_2021_36_MOESM1_ESM.docx]

**Supplementary Online Materials**

To accompany

Emotion naming impedes both cognitive reappraisal and

mindful acceptance strategies of emotion regulation

Nook, Satpute, & Ochsner

Contents

1. Study 1 task instructions
2. Study 2 task instructions
3. Table S1: Emotion words generated by participants
4. Figure S1: Trial-level negative and positive affect rating distributions
5. Analyses of Δ negative and Δ positive affect in Study 1
6. Analyses of negative and positive affect in Study 2

**I. Study 1 Task Instructions**

**Baseline Phase.** For this phase, you will see a series of images. Simply observe each image while it is on the screen. After each image, rate how you feel on the scales provided.

**Look Condition.** For this phase, you will see a series of images. Simply observe each image while it is on the screen. After each image, rate how you feel on the scales provided.

**Name Condition.** For this phase, you will see a series of images. While each image is on the screen, decide what is the most dominant emotion you feel, and say the name of this emotion out loud. Please use only a single word or very short phrase. If it is ever difficult to name what you're feeling, keep trying until you do so or until the image disappears. After each image, rate how you feel on the scales provided.

**Regulate Condition.** For this phase, you will see a series of images. While each image is on the screen, regulate how you feel by creating a story about the image that makes you feel better about it. After each image, rate how you feel on the scales provided.

**Name and Regulate Condition.**  For this phase, you will see a series of images. While each image is on the screen, first decide what is the most dominant emotion you feel, and say the name of this emotion out loud. Please use only a single word or very short phrase. If it is ever difficult to name what you're feeling, keep trying until you do so or until the image disappears. Second, regulate how you feel by creating a story about the image that makes you feel better about it. After each image, rate how you feel on the scales provided.

**II. Study 2 Task Instructions**

**Reinterpret Condition.** For this experiment, you will see a series of images and be asked to rate how they make you feel. Each image will be presented along with two instructions (one after the other) beneath the image. These instructions ask you to interact with the image in a certain way, and we'll teach you what they mean. The three different instructions are “Look,” “Name” and “Regulate.” Even though each trial will involve a series of two instructions, we will teach you what each of these means one by one.

If the word “Look” is presented under the image, simply observe the image naturally, allowing yourself to feel whatever it makes you feel. If the word “Name” is presented under the image, decide what is the most dominant emotion that you feel and say the name of this emotion out loud. Please use only a single word or a very short phrase. If it is ever difficult to name what you're feeling, just keep trying until you do so or until the instruction under the image changes. If the word “Regulate” is presented under the image, try to make yourself feel better about the image by reappraising the scene so that it means something different to you, something that makes you feel better. You can do this by creating a story or context around the image changes how you feel about it. You do not need to say anything out loud for this instruction.

Now we'll teach you how to use the rating scales. After each image, you'll have two rating scales, one asking how positive you feel, and the other asking how negative you feel. The order will vary across trials, so pay attention to which one you're answering. Use the numbers 1-7 to rate how you feel on these scales. Please be as honest as you can.

**Accept Condition.**  Same as *Reinterpret* condition, except “Regulate” instructions were: “If the word “Regulate” is presented under the image, try to make yourself feel better about the image by accepting whatever it makes you feel. Try to remember that whatever you feel is just an emotion - it can not harm you and it will soon pass. Make yourself feel better by acknowledging what you feel and letting it go. You do not need to say anything out loud for this instruction.”

**III. Table S1: Emotion Words Generated by Participants**

| Emotion | Study 1 | Study 2 |  | Emotion | Study 1 | Study 2 |
| --- | --- | --- | --- | --- | --- | --- |
| Abandoned | 0 | 1 |  | Calm | 1 | 2 |
| Abandonment | 0 | 1 |  | Cautious | 0 | 1 |
| Abused | 0 | 1 |  | Caved in | 1 | 0 |
| Abusive | 0 | 1 |  | Chaos | 1 | 1 |
| Acceptance | 0 | 1 |  | Closed in | 0 | 1 |
| Adventurous | 0 | 1 |  | Closed off | 1 | 0 |
| Afraid | 11 | 12 |  | Cold hearted | 0 | 1 |
| Aghast | 0 | 1 |  | Combative | 0 | 1 |
| Alarm | 1 | 0 |  | Comfort | 0 | 1 |
| Alarmed | 1 | 0 |  | Compassion | 2 | 4 |
| Amazed | 2 | 2 |  | Compassionate | 0 | 2 |
| Ambivalent | 3 | 1 |  | Concern | 10 | 2 |
| Amused | 0 | 6 |  | Concerned | 9 | 24 |
| Amusement | 0 | 1 |  | Confined | 1 | 1 |
| Anger | 12 | 39 |  | Confinement | 0 | 1 |
| Angry | 15 | 45 |  | Conflict | 0 | 1 |
| Annoyance | 0 | 1 |  | Confused | 27 | 56 |
| Annoyed | 5 | 2 |  | Confusion | 7 | 8 |
| Anti-religious | 0 | 1 |  | Contaminated | 0 | 1 |
| Anxiety | 5 | 1 |  | Contemplative | 1 | 1 |
| Anxious | 5 | 7 |  | Cool | 2 | 4 |
| Apathetic | 1 | 0 |  | Crazy | 1 | 3 |
| Appalled | 0 | 2 |  | Creeped out | 2 | 1 |
| Apprehensive | 1 | 1 |  | Creepiness | 0 | 1 |
| Arachnophobia | 1 | 0 |  | Creepy | 1 | 3 |
| Arachnophobic | 1 | 0 |  | Cruel | 3 | 1 |
| Ashamed | 0 | 2 |  | Curiosity | 1 | 5 |
| Attack | 0 | 1 |  | Curious | 13 | 20 |
| Attacked | 0 | 4 |  | Cute | 1 | 4 |
| Attacking | 0 | 1 |  | Danger | 1 | 3 |
| Authoritative | 0 | 1 |  | Dangerous | 2 | 4 |
| Awe | 0 | 2 |  | Daring | 0 | 1 |
| Awed | 0 | 1 |  | Dark | 1 | 0 |
| Awesome | 0 | 1 |  | Dead | 0 | 3 |
| Awestruck | 1 | 0 |  | Death | 0 | 6 |
| Awful | 3 | 6 |  | Deathly | 0 | 1 |
| Bad | 7 | 11 |  | Defeated | 0 | 1 |
| Beautiful | 1 | 1 |  | Defensive | 0 | 1 |
| Bitter | 0 | 1 |  | Defiant | 0 | 2 |
| Bleak | 0 | 1 |  | Demoralized | 0 | 1 |
| Bothered | 0 | 3 |  | Depressed | 2 | 13 |
| Boxed in | 1 | 0 |  | Depressing | 1 | 8 |
| Brave | 0 | 1 |  | Depression | 0 | 1 |
| Brutal | 0 | 1 |  | Deserted | 0 | 1 |
| Brutalized | 0 | 1 |  | Desperate | 0 | 1 |
| Bummed | 0 | 1 |  | Destitute | 1 | 0 |
| Emotion | Study 1 | Study 2 |  | Emotion | Study 1 | Study 2 |
| Destroyed | 0 | 1 |  | Empathy | 4 | 2 |
| Destruction | 1 | 0 |  | Endless | 0 | 1 |
| Destructive | 0 | 2 |  | Enraged | 0 | 1 |
| Determination | 0 | 1 |  | Entertained | 0 | 2 |
| Detested | 0 | 1 |  | Excited | 1 | 4 |
| Devastated | 0 | 1 |  | Excitement | 2 | 0 |
| Dirty | 4 | 8 |  | Expletive | 0 | 2 |
| Disappointed | 2 | 15 |  | Exploration | 0 | 1 |
| Disappointment | 2 | 2 |  | Fake | 0 | 1 |
| Disaster | 0 | 1 |  | Fear | 49 | 90 |
| Disastrous | 1 | 1 |  | Fearful | 0 | 1 |
| Discomfort | 0 | 2 |  | Feeble | 0 | 1 |
| Disfigured | 0 | 1 |  | Fierce | 1 | 0 |
| Disgruntled | 0 | 1 |  | Filthy | 0 | 1 |
| Disgust | 48 | 74 |  | Fine | 4 | 6 |
| Disgusted | 47 | 122 |  | Forced | 0 | 1 |
| Disgusting | 6 | 29 |  | Forceful | 0 | 1 |
| Disheartened | 1 | 0 |  | Freaked out | 1 | 4 |
| Disheartening | 0 | 1 |  | Freaky | 0 | 3 |
| Disinterested | 0 | 1 |  | Freedom | 2 | 0 |
| Dislike | 0 | 1 |  | Friendly | 0 | 1 |
| Dismayed | 0 | 1 |  | Friendship | 0 | 1 |
| Despair | 4 | 7 |  | Fright | 1 | 0 |
| Disrespected | 1 | 0 |  | Frightened | 4 | 18 |
| Distanced | 1 | 0 |  | Frightening | 0 | 6 |
| Distaste | 1 | 0 |  | Frustrated | 2 | 5 |
| Distraught | 0 | 3 |  | Frustrating | 1 | 0 |
| Distress | 1 | 0 |  | Fun | 0 | 2 |
| Distressed | 0 | 3 |  | Funny | 1 | 2 |
| Distressing | 1 | 2 |  | Gloomy | 0 | 1 |
| Disturbed | 4 | 18 |  | Good | 1 | 0 |
| Disturbing | 0 | 3 |  | Grateful | 1 | 0 |
| Dominance | 0 | 1 |  | Grief | 0 | 10 |
| Dominated | 0 | 1 |  | Grief stricken | 0 | 1 |
| Don't feel much | 1 | 0 |  | Grimacing | 0 | 1 |
| Don't know | 0 | 6 |  | Grisly | 0 | 1 |
| Don't know what's going on | 2 | 0 |  | Gross | 12 | 50 |
| Don't understand | 0 | 1 |  | Grossed out | 18 | 43 |
| Doubt | 1 | 0 |  | Gruesome | 0 | 1 |
| Doubtful | 0 | 1 |  | Guilty | 1 | 2 |
| Dreadful | 0 | 1 |  | Happiness | 0 | 1 |
| Drowning | 0 | 1 |  | Happy | 1 | 13 |
| Dumb | 1 | 0 |  | Harsh | 0 | 1 |
| Emotion | 0 | 1 |  | Hate | 0 | 3 |
| Emotional | 1 | 0 |  | Hatred | 0 | 1 |
| Empathetic | 0 | 2 |  | Heart wrenching | 1 | 0 |
|  |  |  |  |  |  |  |
| Emotion | Study 1 | Study 2 |  | Emotion | Study 1 | Study 2 |
| Heartbreaking | 0 | 1 |  | Interested | 3 | 10 |
| Heartbroken | 2 | 1 |  | Interesting | 0 | 1 |
| Helpfulness | 1 | 0 |  | Intimidated | 1 | 6 |
| Helping | 0 | 2 |  | Intrigued | 5 | 5 |
| Helping each other | 0 | 1 |  | Invaded | 0 | 1 |
| Helpless | 6 | 1 |  | Itchy | 0 | 1 |
| Helplessness | 1 | 0 |  | Joke | 0 | 1 |
| Hero | 0 | 1 |  | Joy | 0 | 1 |
| Heroic | 0 | 1 |  | Just | 0 | 1 |
| Hope | 2 | 0 |  | Laugh | 0 | 1 |
| Hopeful | 5 | 9 |  | Lonely | 1 | 3 |
| Hopeless | 4 | 4 |  | Loss | 0 | 1 |
| Horrendous | 0 | 1 |  | Love | 0 | 1 |
| Horrible | 5 | 4 |  | Mad | 1 | 2 |
| Horrific | 0 | 1 |  | Malnourished | 0 | 1 |
| Horrified | 8 | 24 |  | Mean | 1 | 1 |
| Horrifying | 0 | 2 |  | Meaningful | 0 | 1 |
| Horror | 9 | 19 |  | Meaningless | 1 | 0 |
| Horrorstricken | 0 | 3 |  | Melancholy | 0 | 1 |
| Humor | 0 | 1 |  | Menacing | 0 | 1 |
| Humored | 0 | 1 |  | Misery | 0 | 1 |
| Hunger | 0 | 2 |  | Mixed | 1 | 0 |
| Hungry | 0 | 3 |  | Moody | 0 | 1 |
| Hurt | 1 | 8 |  | Motivated | 1 | 0 |
| Hurtful | 0 | 1 |  | Mournful | 0 | 2 |
| Icky | 0 | 1 |  | Mourning | 0 | 3 |
| Ignorant | 1 | 0 |  | Nasty | 1 | 4 |
| Ill | 1 | 0 |  | Natural | 0 | 1 |
| Impotence | 0 | 1 |  | Nauseous | 1 | 0 |
| Impotent | 0 | 1 |  | Negative | 5 | 0 |
| Impressed | 1 | 2 |  | Nervous | 1 | 4 |
| Impressed by cruelty | 1 | 0 |  | Neutral | 7 | 14 |
| Impressive | 0 | 1 |  | Neutrality | 2 | 0 |
| Imprisoned | 1 | 1 |  | No emotions | 1 | 0 |
| In awe | 1 | 0 |  | No feelings | 3 | 1 |
| In search of an explanation | 1 | 0 |  | Nonchalant | 1 | 0 |
| Incisive | 0 | 1 |  | Normal | 4 | 2 |
| Indifference | 2 | 1 |  | Nostalgic | 1 | 0 |
| Indifferent | 8 | 10 |  | Not good | 1 | 0 |
| Infected | 0 | 1 |  | Not hungry | 1 | 0 |
| Inhumane | 0 | 1 |  | Not OK | 0 | 1 |
| Injured | 0 | 1 |  | Not scared | 0 | 4 |
| Innocence | 0 | 1 |  | Not strong | 0 | 1 |
| Inquisitive | 0 | 1 |  | Not sure | 2 | 0 |
| Inspired | 0 | 1 |  | Nothing | 9 | 13 |
| Interest | 2 | 2 |  | Offended | 0 | 3 |
|  |  |  |  |  |  |  |
| Emotion | Study 1 | Study 2 |  | Emotion | Study 1 | Study 2 |
| OK | 4 | 7 |  | Relief | 0 | 2 |
| Oppressed | 0 | 1 |  | Relieved | 3 | 2 |
| Optimism | 1 | 0 |  | Remorse | 1 | 1 |
| Optimistic | 1 | 0 |  | Repulsed | 0 | 3 |
| Outraged | 0 | 2 |  | Resistance | 0 | 1 |
| Overwhelmed | 1 | 0 |  | Revolted | 0 | 1 |
| Ow | 0 | 1 |  | Revulsion | 2 | 0 |
| Pain | 3 | 27 |  | Rotting | 0 | 1 |
| Pained | 2 | 1 |  | Sad | 89 | 252 |
| Painful | 5 | 6 |  | Saddened | 4 | 2 |
| Panic | 1 | 2 |  | Saddening | 0 | 1 |
| Panicked | 2 | 1 |  | Sadness | 18 | 69 |
| Peace | 0 | 1 |  | Safe | 1 | 0 |
| Peaceful | 1 | 0 |  | Safety | 0 | 1 |
| Perplexed | 1 | 0 |  | Scandalized | 0 | 1 |
| Petrified | 0 | 2 |  | Scared | 45 | 176 |
| Petrifying | 0 | 1 |  | Scary | 1 | 43 |
| Phobia | 0 | 1 |  | Screaming | 0 | 1 |
| Pitiful | 0 | 3 |  | Seething | 0 | 1 |
| Pity | 39 | 24 |  | Serious | 0 | 1 |
| Playful | 0 | 1 |  | Shame | 3 | 2 |
| Poison | 1 | 0 |  | Shock | 6 | 11 |
| Poor | 0 | 1 |  | Shocked | 7 | 22 |
| Power | 0 | 1 |  | Shocking | 0 | 1 |
| Powerless | 1 | 1 |  | Sick | 3 | 7 |
| Prayerful | 0 | 1 |  | Sickening | 0 | 1 |
| Pretty | 1 | 0 |  | Sickness | 0 | 1 |
| Pride | 0 | 1 |  | Silly | 0 | 5 |
| Protection | 0 | 2 |  | Skin crawley | 1 | 0 |
| Protective | 0 | 1 |  | Small | 1 | 0 |
| Proud | 0 | 1 |  | So so | 3 | 0 |
| Psychic | 1 | 0 |  | Solemn | 0 | 1 |
| Punished | 0 | 1 |  | Solemnity | 0 | 1 |
| Punishment | 0 | 1 |  | Sorrow | 3 | 8 |
| Put off | 1 | 1 |  | Sorry | 5 | 11 |
| Puzzled | 1 | 1 |  | Squeamish | 1 | 0 |
| Questionable | 1 | 0 |  | Squeamishness | 1 | 0 |
| Racist | 0 | 1 |  | Strange | 1 | 0 |
| Rattled | 1 | 0 |  | Strength | 0 | 2 |
| Ready to attack | 0 | 1 |  | Stressed | 0 | 1 |
| Reality | 1 | 0 |  | Stressed out | 1 | 0 |
| Reality of Life | 1 | 0 |  | Struggling | 1 | 1 |
| Regret | 0 | 3 |  | Stupid | 1 | 1 |
| Regretful | 0 | 2 |  | Stupidity | 0 | 1 |
| Rejoice | 0 | 1 |  | Success | 0 | 1 |
| Relaxed | 0 | 1 |  | Sudden | 0 | 1 |

| Emotion | Study 1 | Study 2 |  | Emotion | Study 1 | Study 2 |
| --- | --- | --- | --- | --- | --- | --- |
| Suffering | 0 | 1 |  | Unnecessary | 0 | 1 |
| Suffocating | 0 | 1 |  | Unphased | 0 | 2 |
| Sunken | 0 | 1 |  | Unpleasant | 2 | 0 |
| Surprise | 2 | 4 |  | Unsafe | 0 | 1 |
| Surprised | 1 | 12 |  | Unsettling | 0 | 1 |
| Sweet | 0 | 1 |  | Unsettled | 0 | 4 |
| Sympathetic | 4 | 20 |  | Unsure | 3 | 2 |
| Sympathy | 6 | 13 |  | Untidy | 1 | 0 |
| Taken aback | 1 | 1 |  | Upset | 13 | 44 |
| Targeted | 1 | 0 |  | Upsetting | 0 | 4 |
| Teary | 0 | 1 |  | Urgency | 1 | 0 |
| Terrible | 8 | 1 |  | Useless | 1 | 0 |
| Terrific | 0 | 1 |  | Usual | 1 | 1 |
| Terrified | 11 | 27 |  | Venomous | 0 | 1 |
| Terrifying | 0 | 8 |  | Vicious | 0 | 2 |
| Terror | 2 | 11 |  | Victimized | 0 | 1 |
| Thankful | 0 | 1 |  | Vindictive | 0 | 1 |
| Thirsty | 0 | 1 |  | Violated | 0 | 1 |
| Threatened | 4 | 10 |  | Violence | 0 | 2 |
| Threatening | 1 | 1 |  | Violent | 0 | 7 |
| Tight | 0 | 1 |  | Want to throw up | 1 | 1 |
| Tired | 1 | 2 |  | Wanting to jump | 0 | 1 |
| Together | 0 | 1 |  | Wanting to yell | 0 | 1 |
| Torture | 0 | 2 |  | Wasteful | 0 | 1 |
| Tortured | 0 | 1 |  | Weakness | 0 | 1 |
| Touching | 1 | 0 |  | Weird | 2 | 6 |
| Tragedy | 0 | 3 |  | Weirded out | 1 | 1 |
| Tragic | 2 | 12 |  | What | 0 | 1 |
| Trapped | 4 | 3 |  | Wonder | 1 | 0 |
| Trashy | 0 | 2 |  | Wondering | 1 | 1 |
| Tyranny | 0 | 1 |  | Worried | 16 | 40 |
| Ugly | 0 | 1 |  | Worry | 8 | 3 |
| Unaffected | 0 | 7 |  | Worst | 1 | 0 |
| Unaware | 0 | 1 |  | Yuck | 1 | 5 |
| Unclean | 0 | 1 |  |  |  |  |
| Uncomfortable | 9 | 6 |  |  |  |  |
| Unconcerned | 0 | 1 |  |  |  |  |
| Understanding | 0 | 2 |  |  |  |  |
| Unease | 0 | 2 |  |  |  |  |
| Uneasy | 0 | 2 |  |  |  |  |
| Unengaged | 0 | 1 |  |  |  |  |
| Unfair | 0 | 4 |  |  |  |  |
| Unfairness | 1 | 0 |  |  |  |  |
| Unfortunate | 2 | 5 |  |  |  |  |
| Unhappy | 1 | 0 |  |  |  |  |
| Uninterested | 0 | 1 |  |  |  |  |

**IV. Figure S1: Negative and Positive Affect Rating Distributions**


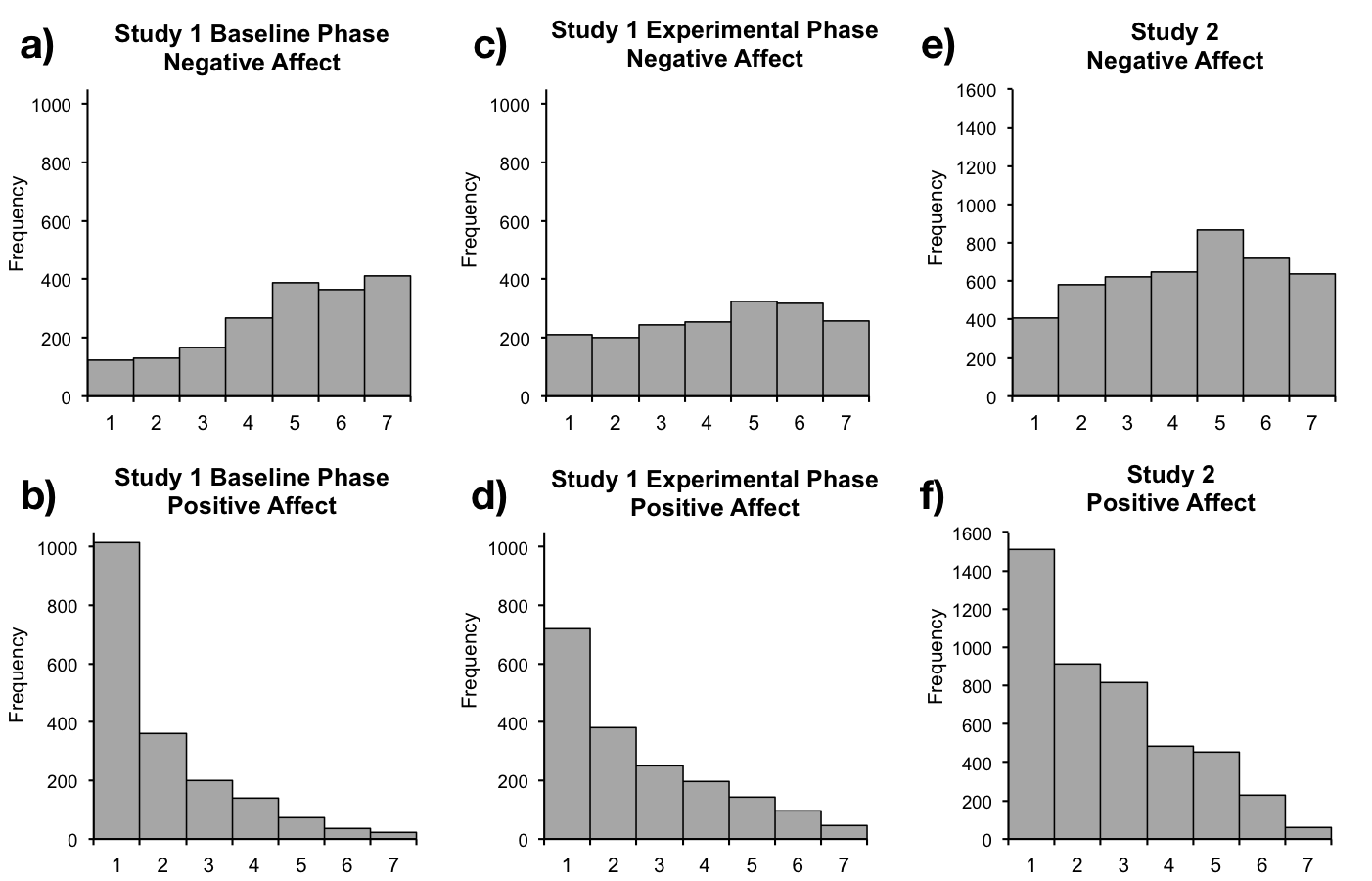


**Fig S1.** Histograms of trial-level negative and positive affect ratings for each study. Note how positive affect ratings clustered at floor. This is likely due to the fact that all images were negative. This restricted range in positive affect ratings likely reduced the trial-level correlations between positive and negative affect ratings. Even though trial-level ratings are not normally distributed, distributions of participant-level means for Δ unpleasant affect, Δ negative affect, and Δ positive affect within each condition of Study 1 approximated normal distributions (Kolmogorov-Smirnov test *p*s > .461), and the same was true for participant-level means of unpleasant, negative, and positive affect in Study 2 (*p*s > .277). Hence, dependent variables met assumptions of statistical tests used in the manuscript.

**V. Analyses of Δ Negative and Δ Positive Affect in Study 1**

**Δ negative affect.** A 2 [Naming] × 2[Regulating] ANOVA revealed a significant main effect of regulating such that participants who regulated their emotions reported feeling less negative affect in the experimental phase (*M* = -1.07, *SD* = 1.04) than participants who did not regulate their emotions (*M* = -0.15, *SD* = 0.51), *F*(1, 76) = 30.85, *p* < .001, η*_p_*^2^ = .29, 90% CI = [.15, .41]. There was also a main effect of naming such that participants who did not name their emotions reported lower negative affect (*M* = -0.94, *SD* = 0.99) than those who did (*M* = -0.28, *SD* = 0.75), *F*(1, 76) = 16.47, *p* < .001, η*_p_*^2^ = .18, 90% CI = [.06, .30]. The interaction between naming and regulating emerged at a trending level of significance for Δ negative affect, *F*(1, 76) = 3.71, *p* = .058, η*_p_*^2^ = .05, 90% CI = [0, .14].

Nonetheless, planned t-tests revealed that naming impeded emotion regulation, as Δ negative affect was lower for participants who *Regulated* their emotions (*M* = -1.56, *SD* = 0.93) compared to those who *Named and Regulated* their emotions (*M* = -0.57, *SD* = 0.92), *t*(38) = -3.38, 95% CI = [-1.56, -0.57], *p* = .002, *d* = -1.07. However, participants who merely *Looked* at images a second time reported less negative affect (*M* = -0.33, *SD* = 0.60) compared to those who *Named* their emotions (*M* = 0.02, *SD* = 0.35), *t*(38) = -2.28, 95% CI = [-0.66, -0.04], *p* = .028, *d* = -0.72. One-way t-tests clarified this result. Participants in the *Look* condition reported significantly reduced negative affect upon their second exposure to the images, *t*(19) = -2.47, 95% CI = [-0.61, -0.05], *p* = .023, *d* = -0.55, whereas participants in the *Name* condition did not, *t*(19) = 0.30, 95% CI = [-0.14, 0.19], *p* = .771, *d* = 0.07. Hence, the difference between these conditions emerged because simply re-viewing negative images reduced negative affect, but naming maintained a consistent level of negative affect. Unsurprisingly, participants in both the *Regulate* condition, *t*(19) = -7.54, 95% CI = [-1.99, -1.13], *p* < .001, *d* = -1.69, and *Name and Regulate* condition, *t*(19) = -2.81, 95% CI = [-1.00, -0.15], *p* = .011, *d* = -0.63, reported significantly reduced negative affect in experimental trials, compared to baseline trials.

**Δ positive affect.** There was a significant main effect of regulating such that participants who regulated their emotions reported feeling more positive affect (*M* = 1.22, *SD* = 1.07) compared to those who did not regulate their emotions (*M* = -0.13, *SD* = 0.39), *F*(1, 76) = 70.87, *p* < .001, η*_p_*^2^ = .48, 90% CI = [.34, .58], and participants who did not name their emotions reported higher positive affect (*M* = 0.77, *SD* = 1.26) than those who did (*M* = 0.33, *SD* = 0.74), *F*(1, 76) = 7.67, *p* = .007, η*_p_*^2^ = .09, 90% CI = [.01, .20]. These effects were qualified by a significant interaction between naming and regulating, *F*(1, 76) = 14.96, *p* < .001, η*_p_*^2^ = .16, 90% CI = [.06, .29].

T-tests again revealed that naming impeded regulation: Δ positive affect was greater for participants who *Regulated* their emotions (*M* = 1.76, *SD* = 1.00) than for those who *Named and Regulated* their emotions (*M* = 0.69, *SD* = 0.87), *t*(38) = 3.58, 95% CI = [0.46, 1.66], *p* < .001, *d* = 1.13, indicating that participants were better at boosting positive affect through reappraisal if they did *not* first name their emotions. Additionally, participants who merely *Looked* at images a second time reported similar changes in positive affect (*M* = -0.21, *SD* = 0.46) compared to those who *Named* their emotions (*M* = -0.04, *SD* = 0.28), *t*(38) = -1.45, 95% CI = [-0.42, 0.07], *p* = .154, *d* = -0.46. One-way t-tests revealed that participants in the *Look* condition showed a trending decrease in positive affect upon their second exposure to the images, *t*(19) = -2.06, 95% CI = [-0.43, 0.003], *p* = .053, *d* = -0.46. Participants in the *Name* condition did not change their positive affect from baseline to experimental phase, *t*(19) = -0.60, 95% CI = [-0.17, 0.09], *p* = .556, *d* = -0.13. Participants in both the *Regulate* condition, *t*(19) = 7.83, 95% CI = [1.29, 2.23], *p* < .001, *d* = 1.75, and *Name and Regulate* condition, *t*(19) = 3.56, 95% CI = [0.28, 1.10], *p* = .002, *d* = 0.79, reported significantly increased positive affect in the experimental phase.

**Summary.** Analyses of negative and positive affect revealed patterns largely consistent with analyses of unpleasant affect. In particular, regulation was less successful when paired with emotion naming. However, there were three differences in these analyses compared to analyses of Δ unpleasant affect. First, the interaction between naming and regulating emerged only at a statistical trend for Δ negative affect (*p* = .058). Second, participants in the *Look* condition reported significantly less negative affect and less positive affect (at a trending level of significance, *p* = .053) in the experimental phase compared to baseline. This suggests that mere re-exposure to negative images reduced their affective impact. However, participants in the *Name* condition showed no significant changes in either positive or negative affect from baseline to experimental phase, a result that supports the claim that naming “crystalizes” emotion. Third, this difference produced a small but significant difference in negative affect between the *Look* and *Name* conditions.

**VI. Analyses of Negative and Positive Affect in Study 2**

**Negative affect.** A 2 [Naming] × 2[Regulating] × 2[Regulation Strategy] ANOVA revealed a main effect of regulation on negative affect ratings *F*(1,58) = 32.53, *p* < .001, η*_p_*^2^ = .36, 90% CI = [.20, .49]. Participants reported significantly lower negative affect after regulating their emotions (*M* = 4.07, *SD* = 1.09) compared to not regulating (*M* = 4.53, *SD* = 1.05). There was also a main effect of naming such that participants felt less negative affect on trials when they did not name their emotions (*M* = 4.19, *SD* = 1.08) compared to when they did name their emotions (*M* = 4.42, *SD* = 1.10), *F*(1,58) = 19.17, *p* < .001, η*_p_*^2^ = .25, 90% CI = [.10, .39]. However, these main effects were again qualified by a significant interaction between naming and regulating, *F*(1,58) = 9.51, *p* = .003, η*_p_*^2^ = .14, 90% CI = [.03, .28].

There was not a main effect of emotion regulation strategy, *F*(1,58) = 0.76, *p* = .387, η*_p_*^2^ = .01, 90% CI = [0, .10], or an interaction between regulation strategy and naming, *F*(1,58) = 0.79, *p* = .377, η*_p_*^2^ = .01, 90% CI = [0, .10] on negative affect. The interaction between regulating and emotion regulation strategy was also significant for negative affect, *F*(1,58) = 4.26, *p* = .043, η*_p_*^2^ = .07, 90% CI = [.001, .19]. There again was no 3-way interaction between naming, regulating, and emotion regulation strategy, *F*(1,58) = 1.99, *p* = .163, η*_p_*^2^ = .03, 90% CI = [0, .14].

Paired-samples t-tests demonstrated that negative affect ratings were significantly lower following *Regulate* trials than the *Name and Regulate* trials, both within the *Reinterpret* condition, *t*(28) = -4.05, *p* < .001, 95% CI = [-0.55, -0.18], *d* = -0.75, and the *Accept* condition, *t*(30) = -3.34, *p* = .002, 95% CI = [-0.55, -0.13], *d* = -0.60. Comparing *Look* and *Name* conditions revealed that naming emotions in the *Reinterpret* condition did not significantly affect negative affect ratings, *t*(28) = 0.04, *p* = .969, 95% CI = [-0.16, 0.16], *d* = 0.007, whereas it appeared to intensify negative affect in the *Accept* condition, *t*(30) = -2.07, *p* = .047, 95% CI = [-0.41, -0.003], *d* = -0.37.

**Positive affect.**  We observed a main effect of regulation on positive affect ratings *F*(1,58) = 27.01, *p* < .001, η*_p_*^2^ = .32, 90% CI = [.16, .45] such that participants reported significantly higher positive affect when regulating their emotions (*M* = 2.83, *SD* = 1.10) compared to not regulating (*M* = 2.40, *SD* = 0.91). There was also a main effect of naming such that participants felt greater positive affect on trials when they did not name their emotions (*M* = 2.66, *SD* = 1.05) compared to when they did name their emotions (*M* = 2.57, *SD* = 1.01), *F*(1,58) = 4.78, *p* = .033, η*_p_*^2^ = .08, 90% CI = [.003, .20]. However, these main effects were again qualified by a significant interaction between naming and regulating, *F*(1,58) = 4.20, *p* = .045, η*_p_*^2^ = .07, 90% CI = [.001, .19].

Emotion regulation strategy did not significantly impact positive affect ratings, *F*(1,58) = 1.02, *p* = .316, η*_p_*^2^ = .02, 90% CI = [0, .11], nor did regulation strategy interact with naming, *F*(1,58) = 0.61, *p* = .439, η*_p_*^2^ = .01, 90% CI = [0, .09]. The interaction between regulating and emotion regulation strategy was also significant for positive affect, *F*(1,58) = 7.83, *p* = .007, η*_p_*^2^ = .12, 90% CI = [.02, .25]. However, there again was no 3-way interaction between naming, regulating, and emotion regulation strategy for positive affect, *F*(1,58) = 0.01, *p* = .922, η*_p_*^2^ < .001, 90% CI = [0, .01].

Condition-wise comparisons revealed that positive affect ratings were higher in *Regulate* trials compared to *Name and Regulate* trials. This difference was trending within the *Reinterpret* condition, *t*(28) = 1.85, *p* = .075, 95% CI = [-0.02, 0.31], *d* = 0.34, and significant within the *Accept* condition, *t*(30) = 2.44, *p* = .021, 95% CI = [0.03, 0.37], *d* = 0.44. Naming emotions did not influence positive affect: There were no significant differences between positive affect ratings in *Look* and *Name* conditions in either the *Reinterpret*, *t*(28) = -0.35, *p* = .728, 95% CI = [-0.23, 0.17], *d* = -0.07, or *Accept* conditions, *t*(30) = 0.53, *p* = .599, 95% CI = [-0.10, 0.18], *d* = 0.10.

**Summary.** These results mirror the combined unpleasant affect analyses with two exceptions: (i) negative affect was significantly higher in *Name* trials compared to *Look* trials for participants in the *Accept* condition, (ii) there was only a trending difference in positive affect between *Regulate* and *Name and Regulate* trials in the *Reinterpret* condition.
